# Detection technologies and metabolic profiling of bile acids: a comprehensive review

**DOI:** 10.1186/s12944-018-0774-9

**Published:** 2018-05-23

**Authors:** Yanan Liu, Zhihui Rong, Dong Xiang, Chengliang Zhang, Dong Liu

**Affiliations:** 1 Department of Pharmacy of Tongji Hospital, Tongji Medical School, Huazhong Science and Technology University, Wuhan, 430030 China; 2 Department of Paediatrics of Tongji Hospital, Tongji Medical School, Huazhong Science and Technology University, Wuhan, 430030 China

**Keywords:** Bile acids, Detection technologies, Liquid chromatography-mass spectrometry, Metabolic profiling, Metabolic disease

## Abstract

Bile acids (BAs) are important regulatory factors of life activities, which are involved in the regulation of glucose, lipid and energy metabolisms, and closely associated with intestinal hormones, microbiotas and energy balance. BAs abnormalities easily lead to inflammation and metabolic diseases, in turn, the progress of diseases could influence characteristics of BAs. Therefore, accurate detection of BAs contents is of great significance to disease prevention, diagnosis and treatment. At present, the most widely used enzymatic method in clinical practice is applicable to the detection of total bile acid (TBA). In laboratory research, different types of BAs can be accurately separated and quantified by liquid chromatography-mass spectrometry (LC-MS). The metabolic profiling of BAs based on detection technologies can completely and accurately monitor their types and contents, playing a crucial role in disease prevention, diagnosis and treatment. We herein reviewed the main detection technologies of BAs and the application of metabolic profiling in related diseases in recent years.

## Background

Bile acids (BAs) are a kind of cholanic acids synthesized from cholesterol in the liver [1]. They are closely related to glucose, lipid, cholesterol and drug metabolisms. BAs also maintain their own synthesis, metabolism and homeostasis by regulating endogenous nuclear receptors such as farnesoid X receptor (FXR) and related pathways. Besides, BAs are involved in the regulation of G-protein-coupled BA receptor (TGR5), which play key roles in stimulating energy metabolism, reducing liver and intestinal inflammations as well as increasing insulin sensitivity [2].

As important regulatory factors of life activities, the disorders of in vivo processes and regulation of BAs easily lead to inflammation, metabolic diseases and other symptoms. In turn, liver and intestinal diseases are often accompanied by systemic cycling disorders of BAs [3, 4], so their characteristics in blood and tissues can indirectly indicate disease states. Based on these, BAs in blood and tissues, especially those in the intestine and liver, have been chosen as biomarkers for many diseases [5], so their separation and quantification have been spotlighted. BAs detection technologies have been researched for decades, as crucial strategies for monitoring their types and contents. Due to similar chemical structures of BAs, the sensitivity and selectivity of detection have become the bottleneck [6]. Since the middle of twentieth century, the detection of BAs has evolved from simple qualitative analysis to accurate quantification, and from the total content to the extraction, separation and quantification of a specific BA, accompanied by simplification of sample processing steps and improvement of sensitivity. Currently, the most widely used enzymatic method in clinical practice is mainly applicable to the detection of total bile acid (TBA). Meanwhile, different types of BAs can be accurately separated and quantified by liquid chromatography-mass spectrometry (LC-MS), as the main analytical method in most studies.

The metabolic profiling of BAs (MPBA) is quantification of BAs and their metabolites based on the theory of metabolomics. It is a modern analytical strategy that guides and assists the diagnosis of BAs metabolic pathway changes, which can reveal their synthesis and the entire metabolic process comprehensively, thoroughly and systematically. MPBA changes can characterize those in the contents of various BAs in different physiological or pathological environments, thereby being an important evidence for disease diagnosis and treatment. With continuous improvement of BAs detection technologies, MPBA has been increasingly used in studies on metabolic, liver and intestinal diseases. We herein reviewed recent studies on the main detection technologies of BAs and the MPBA changes and applications in diseases.

## Cycling of BAs

BAs are synergistically produced by endogenous metabolic and symbiotic intestinal microbiotas. The process is associated with the catalytic oxidation of cholesterol in the liver and the transformation of intestinal microbiota. Catalytic enzymes are located in the cytoplasm, microsomes, mitochondria and peroxisomes successively. BAs are synthesized via two pathways. The classical pathway is initiated by cytochrome CYP7A1 on the hepatocyte endoplasmic reticulum, which catalyzes cholesterol into 7α-hydroxycholesterol, producing 7α-hydroxy-4-cholesten-3-one (C4) under the catalysis of 3β-hydroxy-Δ^5^-C_27_-hydroxysteroid dehydrogenase in microsomes. Catalyzed by CYP8B1, cholic acid (CA) is finally generated after a series of reactions. Without CYP8B1 metabolism, C4 is eventually metabolized into chenodeoxycholic acid (CDCA). CYP7A1 is the rate-limiting enzyme of this process, the expression of which is regulated by CA level through a negative feedback. In mice liver, most of CDCA is converted to α-muricholic acid (α-MCA) and β-MCA [7]. There is also an alternative synthetic pathway, producing CDCA under the catalysis of CYP27A1 and CYP7B1 [1]. The classic pathway dominates in the synthesis of BAs, with the alternative pathway accounting for less than 10% of TBA under normal human physiological conditions [8]. However, the alternative pathway may be up-regulated when the classic one is obstructed, as the main biosynthetic pathway of BAs in patients with liver diseases [9].

**Fig. 1:**
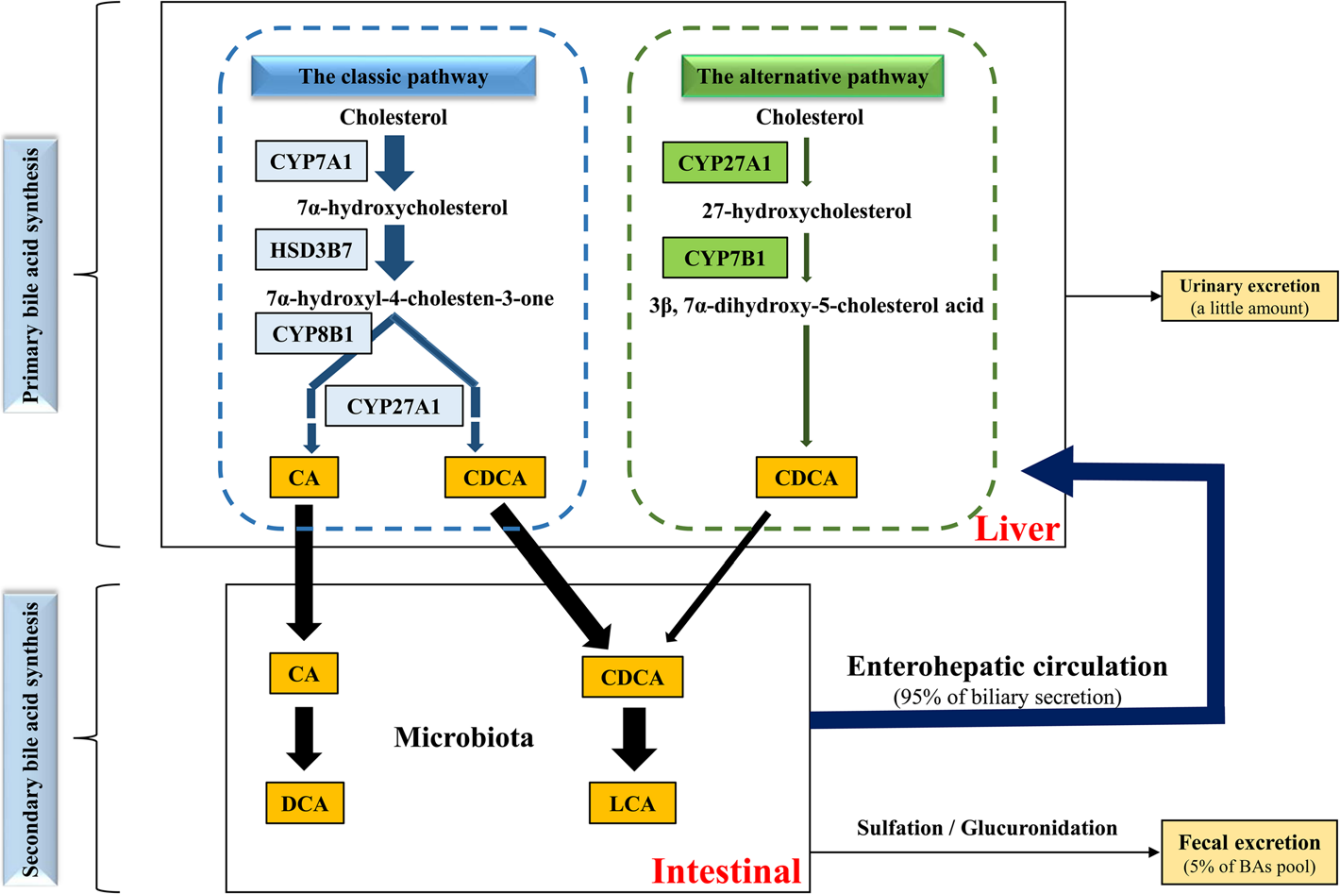
Synthesis and metabolism of common bile acids in human. Two major biosynthetic pathways of bile acids are shown. The classic pathway accounts for over 90% of total bile acids in human. Cholesterol is converted to 7α-hydroxycholesterol by rate-limiting enzyme CYP7A1. Then 7α-hydroxycholesterol is converted to 7a-hydroxy-4-cholesten-3-one (C4) by HSD3B7. Under CYP8B1 and CYP27A1, C4 is converted to CA, and without CYP8B1, C4 is eventually converted to CDCA. In the alternative pathway, cholesterol is first converted to 27-hydroxycholesterol by CYP27A1, and then converted to 3β, 7α-dihydroxy-5-cholestenoic acid by CYP7B1. 3β, 7α-dihydroxy-5-cholestenoic acid is eventually converted to CDCA through a series of reactions. In intestine, CA and CDCA are converted to DCA and LCA through microbiota. About 95% of bile acids are reabsorbed in the intestine and transported back to the liver. This is called the enterohepatic circulation of bile acids. About 5% of the BAs pool is excreted with feces in a day

CA and CDCA, once synthesized, mostly bind taurine or glycine rapidly to form conjugated BAs and then flow into the duodenum from the bile duct. In the intestinal tract, three main reactions are performed by the microbiota: (1) Deconjugation that hydrolyzes taurine and glycine groups on BAs; (2) epimerization; (3) dehydroxylation that acts on free primary BAs, relying on deconjugation as the prerequisite. As a result, primary conjugated BAs are converted into secondary free ones [10, 11], mainly including deoxycholic acid (DCA) and lithocholic acid (LCA). The detailed process is shown in Fig. 1. Primary and secondary BAs undergo reactions such as sulfation, glycosyl esterification and glycosylation after liver and intestinal metabolisms. About 95% of the BAs are reabsorbed through specific transporters at the end of the colon and transported to the liver, with 5% excreted with feces, and a small part excreted with urine. Free BAs in the liver rebind taurine or glycine, and then flow into the intestinal tract through the bile duct. This process is known as the enterohepatic circulation of BAs [9, 12], it ensures efficient utilization of BAs. In mice intestine, CYP3A1 and epimerase convert CDCA into taurohyocholic acid (THCA), ω-muricholic acid (ω-MCA), taurohyodeoxycholic acid (THDCA) and tauroursodeoxycholic acid (TUDCA), of which LCA and ω-MCA are mostly excreted with feces. In a word, the synthesis and metabolism of BAs constitute a complicated cycle that involves many types of structurally similar compounds.

**Fig. 2:**
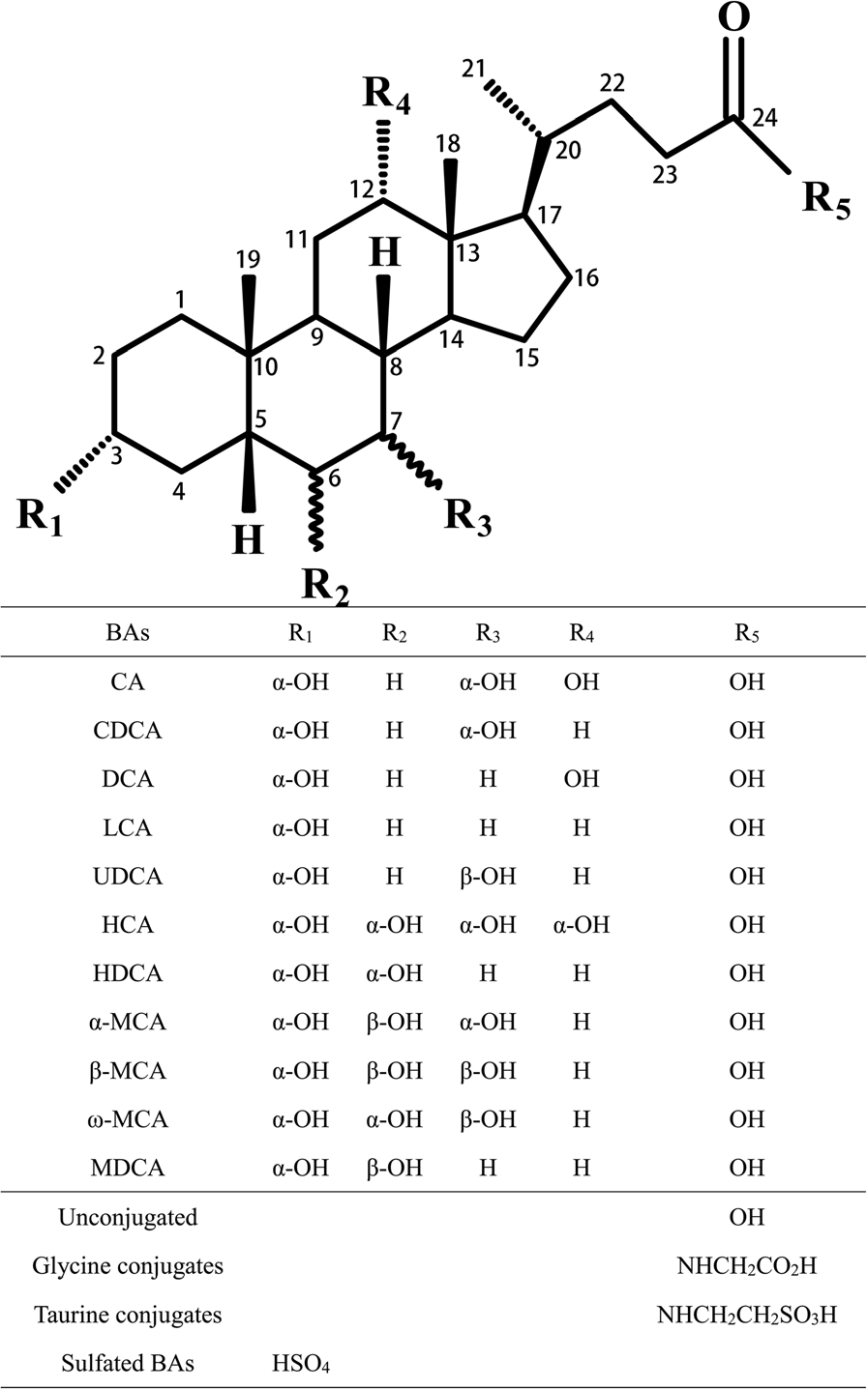
Chemical structure monomer, glycine and taurine conjugates, and sulfated compound of common bile acids [28, 29, 36, 52, 101–103]

BAs have similar structures, i.e. steroids containing carboxyl side chains to which glycine and taurine are usually bound. Human BAs are excreted with urine, mainly after sulfuric acid conjugation which usually occurs on C3-OH. The structures of common BAs are exhibited in Fig. 2.

## Physiological and pathological roles of BAs

BAs can reduce the surface tensions of both oil and water phases, and promote the absorption of fat and lipid-soluble vitamins. In addition, with endogenous signaling molecule-like effects, BAs can regulate FXR, pregnane X receptor (PXR), vitamin D receptor (VDR) and TGR5 to synergistically modulate the homeostasis of BAs and to participate in glucose, lipid, energy and drug metabolisms [2, 13].

BAs are thus involved in the regulation of lipid, glucose and energy metabolisms. The activation of FXR and other nuclear receptors by BAs can inhibit fat synthesis in the liver, decrease very low density lipoprotein (VLDL), and reduce triglyceride (TG) levels [14] by suppressing TG generation as well as promoting TG removal and oxidative decomposition of fatty acids [15]. Likewise, BAs also play essential roles in hepatic glucose metabolism. They inhibit gluconeogenesis and promote glycolysis and glycogen production by activating nuclear receptors, as well as enhance glucose tolerance and insulin sensitivity [16]. Moreover, in pancreatic β cells, BAs can stimulate FXR and TGR5 to facilitate glucose-induced insulin transcription and secretion, thereby regulating liver glucose metabolism [17]. Glucose and fat are two energy sources for organisms. BAs can regulate the metabolisms of lipids and glucose to mediate that of energy. Additionally, decrease in BAs often leads to liver fat accumulation, VLDL overexpression and hypertriglyceridemia, causing obesity and diabetes mellitus.

BAs can also regulate drug metabolism and self-metabolism by activating nuclear receptors such as PXR, constitutive androstane receptor and VDR [8]. For example, LCA can stimulate VDR and accelerate the conversion of toxic BAs into easily excreted compounds so as to reduce their cytotoxicity [18]. Hydrophilic BAs have anti-inflammatory activities, and FXR and TGR5 cascade signaling pathways activated by BAs signals inhibit the nuclear translocation of NF-κB and antagonize NF-κB-induced proinflammatory factors, thereby suppressing the progression of macrophage, intestinal and hepatic inflammations [19, 20]. The activation of intestinal FXR is involved in the regulation of intestinal mucosal barrier and immune response, and can block the overgrowth of intestinal bacteria, repair the injury at the end of the small intestine, and reduce the incidence of liver cancer and colorectal cancer [21].

Diseases and metabolic abnormalities may alter the normal homeostasis of BAs, exhibiting proinflammatory activities. Known hydrophobic BAs, such as DCA and LCA, are strong proinflammatory factors. At over-high concentrations, they show cytotoxicity and lead to DNA damage and cell death, causing damage to the liver, intestinal tract and other tissues [22]. Abnormalities of intestinal BAs induce systemic infection by disrupting the barrier function of the small intestine and promoting the translocation of intestinal bacteria [21]. For example, abnormal increase in the levels of hydrophobic BAs up-regulates the contents of proinflammatory cytokines and NF-κB, changes the composition of intestinal microbiota, increases endotoxin, aggravates the inflammatory response caused by glucose tolerance and insulin resistance, and augments intestinal permeability [23]. High-concentration DCA in the intestinal tract can also promote the excretion of chloride ions, increase intestinal permeability and impede mucosal healing, causing irritable bowel disease [24]. In the liver, DCA can accelerate the senescence of secretory cells, facilitate the generation of tumor-promoting factors, and induce the progression of non-alcoholic steatohepatitis (NASH) and liver cancer [25].

In short, BAs play vital roles in life activities, which, when abnormally regulated, result in diseases, and their contents change correspondingly. Therefore, accurate detection of BAs is beneficial to the diagnosis, monitoring and treatment of diseases.

## BAs detection technologies

Researchers have been devoted to BAs detection technologies for decades, which can mainly be classified into chromatographic and non-chromatographic methods. Chromatographic methods are mainly used for laboratory research, of which thin layer chromatography (TLC) and electrospray ionization (ESI)-MS are primarily applied to qualitative analysis. High performance liquid chromatography (HPLC), gas chromatography (GC)-MS and LC-MS are eligible for both qualitative and quantitative analyses, of which LC-MS is currently the mainstream strategy. These methods have high sensitivity and specificity, together with low detection limit, also with some shortcomings though (Table 1). Non-chromatographic methods are mainly utilized clinically, including the enzymatic method, enzyme-linked immunosorbent assay (ELISA) and nuclear magnetic resonance (NMR). Although they are simple, convenient and rapid, the concomitant high detection limit and low specificity of them are the limitation of usage (Table 1).

**Table 1:** Characteristics of different BAs detection technologies [29, 30, 33, 37, 52, 53, 62, 63, 101, 104, 105]

Method	Advantage	Disadvantage
TLC	Easy operation, low cost; without sample derivatization [30, 104].	Only applicable to qualitative analysis but not samples containing impurities, generally used to detect a mixture of BAs standard substances.
ESI-MS	Without sample derivatization, qualitative analysis is allowed directly according to specific neutral or low-molecular-weight fragment ions [105].	Only applicable to qualitative analysis but not distinguishing of substances with the same parent or fragment ions (e.g. conformational isomers) [29].
HPLC	High sensitivity and specificity, applicable to hardly volatile substances.	Complicated sample treatment that requires derivatization to enhance the UV absorption of BAs, with the efficiency affecting detection results; long detection time that is unsuitable for a large number of samples [33].
GC-MS	High degree of separation giving accurate molecular information, applicable to separation and quantification of mixtures of non-conjugated BAs [105].	Requirement of derivatization that converts conjugated BAs into non-conjugated ones to decrease boiling points, complicated treatment process that is unsuitable for a large number of samples [37].
LC-MS	Short analysis time, low limit of quantification allowing separation of conformational isomers; simple sample treatment, without derivatization, automatic detection suitable for high-throughput analysis [52, 53].	High cost, complicated instrumental operation, mostly applicable to basic research but not clinical use.
Enzymatic method	Routine clinical detection method of TBA, simple operation, low cost, reflecting the overall characteristics of TBA.	Only applicable to C_24_-steroids containing C3-OH which, when substituted, causes detection failure; not applicable to low-content BAs [105].
ELISA	Suitable for a specific BA.	Low accuracy, proneness of antibodies to cross reactions with metabolites or matrices to produce false positive results [62].
NMR	Mainly applicable to a certain type of BAs [63, 101].	Requirement of sample derivatization, complicated operation, failure to detection of a specific BA.

### Chromatographic methods

#### ESI-MS

With simple operation, ESI-MS does not require sample derivatization and is applicable to the detection of unstable BAs, so it is often used for high-throughput analysis. Nowadays, ESI-MS often uses tandem MS (ESI-MS/MS) and low collision energy (< 100 eV). In this mode, structurally simple neutral or low-molecular-weight fragment ions are mainly produced, which can be used for the qualitative analysis of specific conjugated BAs. In the mode of low-energy collision-induced dissociation, Maekawa et al. [26] detected CA, CDCA, DCA, LCA and ursodeoxycholic acid (UDCA) and their conjugated products with glycine, taurine, C3-sulfuric acid, C3-glucuronic acid and C24-glucuronic acid. The negative ion mode of ESI was selected. The m/z values of the characteristic fragments of taurine-conjugated BAs (TCBAs) were 80, 107 and 124, that of glycine-conjugated BAs (GCBAs) was 74, and those of sulfated BAs were 80 and 97 [26]. In this mode, glucose-conjugated BAs were different from amino acid-conjugated ones, producing a fragment ion and neutral fragments. The results were consistent with those reported by Griffiths et al. [27] and Mano et al. [28]. The latter group demonstrated that adding ammonium salts elevated the ionization efficiencies of non-conjugated BAs, GCBAs and TCBAs, and further increased the sensitivity and reproducibility of ESI-MS. Multiple reaction monitoring (MRM) can augment sensitivity by qualitative analysis of specific ion channels.

However, ESI-MS alone suffers from several disadvantages. For instance, competitive ionization restricts the detection limit; identical ion fragments produced by isomers cannot be directly distinguished based on ion channels [29]; free BAs have weak signals; [M-H]^−^ ions of other substances in the matrix may interfere with the detection of target ones. These shortcomings render ESI-MS less commonly used for direct BAs detection, which is usually coupled with GC or LC (4.1.4 and 4.1.5).

#### TLC

At the end of the twentieth century, TLC was used for the qualitative analysis of BAs due to simplicity and low cost, without needing dissociation or derivatization of BAs with high polarity. Nevertheless, this method is not applicable to quantification. For example, a mixture of seven standard substances with different Rf values, including CDCA, DCA, CA, glycocholic acid (GCA), LCA, glycodeoxycholic acid (GDCA) and glycolithocholic acid (GLCA), can be qualitatively analyzed by two-dimensional TLC [30]. Moreover, TLC has also been used to evaluate the lipophilicity of BAs and their conjugated molecules [31, 32].

#### HPLC

HPLC is the most widely used chromatographic separation technology, featuring high sensitivity and specificity. It is suitable for both qualitative and quantitative analyses of BAs. HPLC is generally coupled with UV detector, but BAs themselves have weak UV absorptions that can hardly be detected. Thus, proper sample pretreatment with derivatization is inevitable. Different derivatization methods give various detection wavelengths. For example, Shi et al. [33] detected hydeoxycholic acid (HDCA), CA, CDCA and DCA in *Calculus Bovis Sativus* using nitrophenacyl bromide as the derivatization reagent at the wavelength of 263 nm. Kakiyama et al. [34] performed HPLC for BAs in feces after methylation derivatization of C24-COOH using benzoyl compound. Meanwhile, they separated and detected C_24_BAs including CA, CDCA and DCA at the wavelength of 254 nm. HPLC can also be coupled with fluorescence detector to further raise the sensitivity and selectivity. For instance, rat liver BAs separated by HPLC were treated with the enzymatic method and quantified by using a fluorescence detector. As a result, more types of BAs could be detected, and the detection limit reached 0.02 nmol per 100 mg of liver tissue. However, the long detection time restricts its application currently [35]. Although HPLC has high separation efficiency, it suffers from long injection time and complex process of sample treatment, thus being unsuitable for detecting a large number of samples.

Compared with HPLC, the new-generation ultrafast LC (UFLC) has shorter analysis time as well as higher sensitivity and peak capacity. Si et al. [36] established a method for the detection of 13 BAs in bile within 15 min, without requiring sample derivatization. Besides, selecting acidic phosphates as the mobile phase can simultaneously detect conjugated and free BAs. Compared with LC and HPLC, UFLC has shorter operational time, narrower peak width, lower limit of quantification, together with higher retention capacity and signal to noise ratio. Accordingly, it can be used to detect clinical bile samples.

#### GC-MS and SFC-MS

In early studies, BAs were mostly commonly quantified by GC and GC-MS. GC-MS outweighs other methods mainly in the efficient separation and detection of free BAs mixtures. Compared with LC and HPLC, the GC chromatographic column has a stronger separation ability, revealing more information about molecular structures. Early GC-MS detection was determined by the separation of conjugated and non-conjugated BAs and the conversion of non-conjugated BAs into nonpolar gaseous derivatives. This method can separate and quantify non-conjugated BAs, GCBAs, TCBAs and sulfated BAs.

The sample pretreatment process of GC-MS is complicated, with a different derivatization mechanism from that of HPLC. Since BAs themselves have high boiling points and can hardly be volatilized, it is often necessary to convert conjugated BAs into non-conjugated ones for derivatization. The selection of derivatization methods and reagents evidently affects the parameters and ion channels of GC-MS. Taking LCA, DCA, CDCA, HDCA, CA and UDCA as examples, Tsai et al. [37] evaluated the efficiencies of different derivatization reactions. BAs first react with methanol to produce bile acid methyl ester (A), and then derivatized products are obtained through acetylation (B) or methylsilylation (C). In reaction A, camphorsulfonic acid had the highest catalytic efficiency for the generation of bile acid methyl ester in the ultrasound environment. Compared with acetylation derivatization, methylsilylation derivatization using N,O-bis(trimethylsilyl) trifluoroacetamide proceeded faster under more moderate conditions, with higher efficiency. Chemical ionization ion source was more suitable for BAs detection under these derivatization conditions. By trimethylsilylation etherification using N-methyl-N-(trimethylsilyl) trifluoroacetamide (MSTFA), Matysik and Schmitz [38] obtained CA, CDCA, UDCA, DCA and LCA which had higher signal-to-noise ratios in the presence of electron impact (EI) ion source. For improvement, MSTFA can also be replaced with MSTFA:NH_4_I:dithioerythritol (500:4:2, *v*/*w*/w) using EI ion source and single ion monitoring (SIM), aiming to analyze the contents of seven common BAs in urine [39]. Unlike ESI-MS or LC-MS, the same BA can produce different ion fragments under various experimental conditions owing to the differences between derivatization methods.

GC-MS is applicable to both the detection of BAs in biological samples and the quantification of them in soil. For example, a Soxhlet extractor was used to extract stanols, △^5^-sterols and BAs in soil and geological sediment samples [40], and a continuous liquid-liquid extraction method was used to separate BAs from neutral stanols and sterols. Afterwards, BAs were quantified with SIM by GC-MS after two methylation derivatizations.

Supercritical fluid chromatography (SFC), which incorporates the characteristics of GC and LC, can analyze hardly gasified nonvolatile samples. Meanwhile, this method is more efficient and takes shorter analysis time than LC, as a supplement to GC and LC accordingly. SFC uses supercritical carbon dioxide gas (SCCO_2_) as the mobile phase. SCCO_2_ has low viscosity, and high diffusivity and separation capability [41], the polarity of which changes along with the mixed solvents, so it can separate both hydrophilic and hydrophobic substances. With high resolution and rapid separation, SFC-MS has been used to separate and to quantify 24 kinds of BAs in rat plasma. Compared with GC-MS, SFC-MS is typified by short detection time together with high separation ability and sensitivity [42].

#### LC-MS

##### Characteristics

HPLC or ultra-performance liquid chromatography (UPLC) coupled with MS is the mainstream method to simultaneously detect mixture of BAs, as it can improve the separation of isomers with the same ion fragments. When BAs are detected by LC-MS, aqueous and organic phases containing formic acid or ammonium acetate are often used as the mobile phases for gradient elution to increase the degree of separation. The elution times differ significantly because of different types of LC and BAs awaiting separation, and UPLC usually has shorter elution time and higher separation efficiency. Most studies conducted MS/MS with ESI ion source in the negative ion mode using MRM and SIM to increase the selectivity and sensitivity. LC-MS-based strategies have high accuracies, and their lower limits of quantification (LLOQ) usually reach down to 0.1 ng/mL, most of which are applicable to the detection of different species and samples. Compared with HPLC, LC-MS has higher specificity and shorter detection time, but phosphates should not be used as the mobile phase to prevent from damaging mass spectrometers. Compared with GC-MS and HPLC, LC-MS has quick sample prepared methods without requiring derivatization. Given the requirement of small sample amounts and automatic operation, it is suitable for analyzing samples on a large scale. Nowadays, LC-MS has become the mainstream detection method for BAs.

##### Sample prepared methods

The biological samples for BAs detection mainly include serum, plasma, bile, tissue homogenate, urine and feces, and adipose tissue in some cases. The preparation methods are selected according to sample characteristics, concentration of BAs and interfering substances, matrix effects, etc. When samples are pure and BAs concentrations are high, simple dilution can met the analysis requirement. However, extraction, adsorption and enrichment are in need in the case of complex sample compositions and low BAs concentration. The main purpose of sample treatment is to remove components like proteins, lipids and salts.

In earlier studies, samples were often added a small amount of ammonium hydroxide or sodium hydroxide to attenuate the binding of proteins to BAs, thus facilitating extraction [43]. Nevertheless, under alkaline conditions, ester bonds are subjected to hydrolysis or elimination reaction, leading to errors. At present, protein precipitation, solid phase extraction (SPE) and liquid-liquid extraction are mainly used for treating samples. In some studies, BAs in the feces were extracted by Soxhlet extractor. Notably, some of these methods can also be used to treat samples in other detection technologies mentioned above.

SPE is one of the most selective and widely used methods for enriching and purifying BAs, which can also eliminate the impact of water-soluble salts in samples on mass spectrometer. Variations of conditions such as sample volume and solvent elution power all affect the extraction recovery. Nineteen types of BAs in piglet bile have been extracted by SPE, with the recoveries of 89.1–100.2%. Briefly, bile samples were diluted with pure water, mixed with internal standard and buffer, loaded onto a C18 solid phase extraction column that had been activated by methanol, and rinsed with water and methanol successively. The resulting extracting solution was evaporated under nitrogen and redissolved by methanol before analyzed by LC-MS/MS [44]. Since SPE can simultaneously enrich and purify BAs, and elevate the detection sensitivity, it has been widely used to prepared biological samples such as bile, urine and adipose tissue [45]. SPE is superior to the solvent extraction method primarily in the removal of inorganic salts from samples before direct detection by ESI-MS in the negative ion mode.

Biological samples barely containing interfering substances, such as plasma and serum, are often prepared by the protein precipitation method. This method mostly uses methanol or acetonitrile as the precipitant [46, 47]. In special cases, 5% ammonium hydroxide was also added to enhance protein precipitation. The main processes include centrifugation after vortexing of serum or plasma samples, collection and evaporated with nitrogen, and redissolution with mobile phase into solutions containing BAs for analysis. Compared with SPE and liquid-liquid extraction, protein precipitation is simpler and less time-consuming, as the main preparation method for plasma and serum samples.

In recent years, solid phase microextraction (SPME) has gradually been applied to the extraction and purification of BAs. SPME is suitable for a variety of biological matrices because of better sample purification and elimination of matrix effects, as well as one-step extraction and purification of detection substances [48]. For example, compared to liquid-liquid extraction, fish bile samples prepared by SPME showed good consistency and using less solvents [49]. Boyacı et al. [50] extracted BAs with a modified SPME strategy, referred as thin-film microextraction (TFME). Bessonneau et al. [51] studied bronchoalveolar BAs using TFME with a 50% methanol-activated polyacrylonitrile-C18 film. This method was predominantly affected by desorption time and solvent composition.

Collectively, sample treatment is an important step in BAs detection process. The protein precipitation method, which is simple, rapid and low-cost while using small amounts of solvents, has been widely employed, especially for plasma and serum samples on a large scale. In contrast, biological tissue samples are commonly separated and extracted by SPE. This method plays key roles in reducing impurity interference and matrix effects, enriching low-content substances and raising detection sensitivity.

##### Application of LC-MS

With burgeoning development recently, LC-MS has been given first priority in the accurate detection of BAs in experimental studies. Currently, MPBA is mainly constructed based on LC-MS, and has been applied in medical and biological studies to detect BAs in the liver, intestinal tissues and body fluid of experimental animals together with in human blood, urine and feces [47, 52–56]. For instance, John et al. [53] utilized LC-MS/MS to quickly quantify 34 types of sterols in mouse plasma, urine, gallbladder, liver, feces and adipose tissue, including hydroxyl sterols, primary and secondary BAs, TCBAs and GCBAs, and applied it to the tracing of enterohepatic circulation. Jäntti et al. [52] established a method to detect 33 known BAs and 28 unknown ones in human adipose tissue, which mainly inculded CA, DCA, taurocholic acid (TCA), tauro-β-MCA (Tβ-MCA), GCA, GLCA, GUDGA, glycochenodeoxycholic acid (GCDCA) and GDCA, being different from the main kinds in blood.

Sulfated BAs is one of the BAs metabolic pathways in human urine, which have gradually been highlighted in recent years. Bathena et al. [55] desulfated 15 sulfated BAs in human urine with dioxane before LC-MS/MS. Huang et al. [56] established a detection method without desulfation pretreatment through SPE of sulfated BAs in mouse urine and direct injection thereafter, verifying that sulfation was a secondary pathway for BAs metabolism in mice.

Additionally, LC-MS has also been employed to study BAs in marine animals. Wang et al. [57] reported that the plasma, liver, intestinal tract and gill tissues of lampreys contained species-specific BAs such as PZS and 3KPZS that could be quantified by HSO_4_^−^ fragments and PSDS and PADS based on (HSO_4_^−^)_2_ ones.

### Non-chromatographic methods

#### Enzymatic method

The enzymatic cycling reaction is a routine method for clinically detecting serum TBA. This method can quantify TBA indirectly by UV or fluorescence detection of NADH produced by NAD^+^ in the oxidation reaction of BAs. The reaction mechanisms are as followed.

$$ \mathrm{BAs}+{\mathrm{NAD}}^{+\overset{3\alpha \hbox{-} HSD}{\to }}3\hbox{-} \mathrm{ketosteroid}+\mathrm{NADH} $$

$$ 3-\mathrm{ketosteroid}+\mathrm{NADH}\overset{3\alpha - HSD}{\to}\mathrm{BAs}+{\mathrm{NAD}}^{+} $$

Optimized strategies with simpler reactions and higher sensitivities have been established according to this method. Zhang et al. [58] added NADH, thio-NAD^+^ and genetically engineered 3α-HSD in an enzymatic reaction system at 37 °C. Serum TBA was quantified by measuring the absorbance changes of the product thio-NAD at 405/660 nm per minute, with the detection limit of 0.22 μM and without interference from bilirubin, hemoglobin, ascorbates or lactate dehydrogenase. Zhang et al. [59] improved the enzymatic method by using electrochemical detection, and quantified BAs on the basis of electrical signals generated through oxidation of the product NADH on the surface of screen-printed carbon electrodes. With the detection range of 5.00–400 μM, this method gave similar results to those of the routine enzymatic cycling assay, also being highly sensitive, efficient, low-cost and applicable to clinical use.

Although the 3α-HSD-based enzymatic cycling reaction can be carried out readily, there are still deficiencies, such as the failure to specifically distinguish an individual BA. Besides, BAs are C3-OH-substituted C_24_-steroids, while 3α-HSD can oxidize C3-OH of the C_24_-steroid structure specifically and reversibly. Therefore, this method no longer works if C3-OH is substituted with sulfuric acid or glucuronic acid group. Compared with GC-MS and LC-MS, the enzymatic method is not suitable for detecting low-content BAs owing to high limit of quantification.

#### Elisa

ELISA can detect certain BAs in plasma and in vitro samples specifically, simply and fast, which has been applied for over 30 years. Kobayashi et al. [60] detected sulfated GLCA in 25 human urine samples using monoclonal antibody Ab#37 and ELISA. This method was feasible for diagnosing neonatal biliary atresia, hepatobiliary diseases and hepatitis C. Yu et al. [61] established an ELISA method to detect species-specific PZS in different species of lampreys. Performing this method combination with HPLC, they also monitored the secretion rates of BAs. In addition, ELISA can also be applied to the detection of non-biological samples. Baldofski et al. [62] measured isolithocholic acid (ILA) in water samples using polyclonal antibody ELISA, with the detection range of 0.09–15 μg/L. The method had a high recovery rate but a low accuracy, mainly because the quantification of ILA was inaccurate high by ELISA in some matrices probably due to the cross reactions between antibody and ILA metabolites or matrix. Although ELISA is convenient, it has low specificities for trace BAs, easily giving false positive results.

#### NMR

As a less used method, NMR has mainly been applied to determine the total content of a certain type of BAs. Omkar et al. [63] established a method using ^1^H NMR to simultaneously detect the concentrations of GCBAs, TCBAs, TBA and choline-phospholipids (choline-PLs) in human bile. GCBAs and TCBAs were quantified by the area of characteristic methylene (-CH_2_) peak, and TBA and choline-PLs were quantified with the areas of characteristic methyl (-CH_3_) and trimethylamine (-N^+^(CH_3_)_3_) peaks. Bile samples were derivatized by isotope-labeled TSP (3-(trimethylsilyl) propionic-2,2,3,3-d4 acid sodium salt), with TSP as the standard for quantification and chemical shift. To detect BAs by NMR, samples should be derivatized, so this technology is less commonly applied than the enzymatic method.

## MPBA changes and detection in disease states

Metabolic profiling, as a level of metabolomics research, can quantify a certain type of compounds with similar structures and properties as well as the characteristic metabolites in their metabolic pathways. It is a new method for systematic studies on the changes of metabolite spectra, which is suitable for analyzing various endogenous metabolic components in cells, tissues and other biological samples. Currently, this method is highlighted in disease diagnosis and treatment [64]. MPBA, which is established based on LC-MS, has gradually become a crucial strategy for comprehensively and accurately monitoring the contents of various BAs from different biological samples.

BAs are endogenous metabolites, the metabolic profiles of which differ at different stages of physical development or under different physiological conditions. For example, types and contents of BAs vary in human with different ages [65], or those in different disease states [66]. Additionally, MPBA is affected by therapeutic agents such as BAs chelators [67, 68]. Therefore, it is of great significance to the prevention, diagnosis and treatment of a certain disease by monitoring the content changes of a related BA. This section reviews the application and significance of MPBA to related diseases in recent years.

### Liver diseases

As endogenous signaling molecules, BAs can regulate the metabolic pathways and energy balances of lipids and saccharides in the liver and surrounding tissues. The liver, gallbladder and intestine synergistically participate in maintaining the homeostasis of BAs in human body, and only small amounts of BAs can enter the blood circulation in normal states. Liver or biliary tract diseases can cause metabolic disorders of BAs, leading to significant changes in the compositions and contents of serum BAs [69]. Thus, it is possible to diagnose liver diseases by monitoring the characteristics of MPBA in human body.

Sugita et al. [70] compared the plasma levels of 16 BAs in 150 patients with liver diseases and 46 normal subjects. The levels of UDCA and glycoursodeoxycholic acid (GUDCA) in patients with alcoholic liver disease exceeded those in patients with hepatitis, but patients with cholestasis had lower levels of DCA and UDCA than those of hepatitis ones. The serum levels of DCA and LCA in NASH patients were higher than those of the normal group before and after food intake, and the content of conjugated LCA decreased and the cytotoxicity increased after intake of high-fat diet [71]. Sarafian et al. [47] compared 145 BAs in the plasma of normal subjects and patients with liver cirrhosis and liver failure. The levels of TUDCA, TCA, GUDCA, glycohyodeoxycholic acid (GHDCA), GCA and GCDCA significantly rose in patients with liver diseases, whereas those of GCDCA and GDCA dropped. Moreover, the contents of sulfated BAs in the urine of patients with primary biliary cirrhosis were not affected by diet, which could reflect the degree of early liver fibrosis more accurately than TBA content [72]. These studies have provided valuable evidence for the metabolism of BAs and the clinical diagnosis and treatment of liver diseases.

Intrahepatic cholestasis of pregnancy (ICP) is a severe pregnancy-specific disease that can be characterized by serum MPBA. The serum levels of TCA, GCA, taurochenodeoxycholic acid (TCDCA), GCDCA, tauroursodeoxycholic acid (TDCA) and TUDCA in pregnant women with severe ICP, especially the former two, increased compared with those of healthy volunteers and mild patients [73]. Accordingly, the concentration changes of serum primary BAs may be biomarkers for the diagnosis of ICP. It has also been reported that except for significant increase of some BAs such as CA and TCBAs, the serum proportion of CDCA and secondary BAs like DCA and LCA all plummeted, which however, increased with the progression of ICP [74]. Besides TBA content, increase in the ratios of CA/CDCA and TCBAs/GCBAs can also be used as an index for ICP diagnosis, and the levels of TCBAs can be reduced by administration of UDCA [75]. In addition to serum MPBA, urinary MPBA can also be used as a diagnostic index for ICP, which changes more significantly upon ICP, because the detection specificity for sulfated BAs in urine surpasses that for non-sulfated ones [76].

Abnormal elevation of BAs also reflects drug hepatotoxicity [77]. Guo et al. [78] observed the hepatotoxicity of acetaminophen and its metabolites against 48 volunteers, and found that the plasma levels of four primary BAs (TCA, GCA, TCDCA, GCDCA) rose with the progression of liver injury, which may be chosen as early biomarkers for acetaminophen-induced liver injury.

Yang et al. monitored the blood MPBA of cholestatic mice, and found that administration of Danning tablets corrected the abnormal levels of TCA, THDCA, TCDCA and TUDCA [79]. Hence, MPBA can be used for not only the diagnosis and typing of liver diseases, but also the evaluation of treatment outcomes.

### Gastrointestinal diseases

The intestinal tract, as one of the main sites of BAs metabolism, is closely related with MPBA. Gastrointestinal diseases, such as gastric cancer, enteritis and diarrhea, can cause abnormal enterohepatic circulation of BAs and change the characteristics of MPBA. Therefore, analyzing MPBA as a whole is conducive to the diagnosis of gastrointestinal diseases. Recent studies have confirmed that DCA induced cell apoptosis and promoted the carcinomatous changes of gastric mucosal cells, eventually triggering gastric cancer [80]. According to the urine MPBA of patients with gastric cancer, 16 metabolites, including DCA and GCA, can be employed as biomarkers for diagnosis and typing [81, 82].

BAs accumulation in colon tissue causes cytotoxicity and inflammation thereafter, and the cytotoxicities of non-conjugated BAs are higher than those of conjugated ones. The blood TBA content of colitis mice is lower than that of normal ones, but the contents in colon tissues follow an opposite trend. Moreover, the concentrations of non-conjugated BAs increase in the intestinal tract of colitis mice [83].

Intestinal diseases are closely associated with intestinal microorganisms which evidently interact with MPBA. Intestinal bacteria contribute to the conversion of primary BAs into secondary ones, which, when change, usually induce abnormalities in the contents of secondary BAs, conjugated ones in particular, thus altering the bile acid pool [71]. Furthermore, BAs can also change the intestinal microbial composition through direct or indirect antimicrobial effects [84]. For instance, when human MPBA changes due to disturbance of the intestinal microbiota upon diarrhea, the DCA concentration in intestinal tissue and intestinal fluid significantly rise, managing to inhibit the disorders as a homeostatic regulation mechanism [85].

### Metabolic diseases

BAs participate in not only the in vivo digestion and absorption of lipids, but also the regulation of energy metabolism balance via multiple signaling pathways. Obesity is caused by the imbalance of energy metabolism. Exogenous administration of BAs can activate TGR5 and facilitate the energy consumption of brown adipose tissue in mice, thereby preventing high-fat diet-induced obesity and type II diabetes mellitus [86]. The blood glucose levels of patients with type 2 diabetes mellitus can be controlled after gastric bypass surgery, with the serum levels of non-conjugated BAs (CA, DCA and CDCA) significantly rising, indicating that changes in BAs composition may be closely related with glucose metabolism [87]. Besides, orally administering obese C57 mice with FXR-specific agonist can induce the expression of fibroblast growth factor 15 (FGF15), decrease body weight and the phenotype of metabolic deterioration, and alleviate systemic inflammation, accompanied by changes of BAs composition, such as decrease in the proportion of TCA and increase in that of DCA [88].

Gestational diabetes mellitus (GDM) is a manifestation of glucose intolerance during pregnancy. GDM women show severe dyslipidemia, centripetal obesity and insulin resistance, even suffering from postpartum type II diabetes mellitus as the disease progresses [89]. As indicated by MPBA of 38 serum samples of GDM patients, a free BA containing two hydroxyl groups can be used as a marker for distinguishing from ICP, which may be murodeoxycholic acid (MDCA). Compared with the normal group, the β-MCA content significantly increases, also as a biomarker for GDM diagnosis. Additionally, the contents of other six sulfated BAs are also raised significantly [90]. Sulfation can increase the water solubility of BAs, reduce cytotoxicity, and accelerate the discharge of BAs with urine. Therefore, the increase of sulfated BAs in serum of GDM patients might be a protection mechanism for abnormal elevation of BAs concentration.

MPBA may also change during the treatment of metabolic diseases. Colesevelam, as a second-generation BAs chelator, is commonly combined with statins or antidiabetic drugs to control blood glucose. After treatment of type II mellitus diabetes with colesevelam, CA synthesis is enhanced, but the contents of CDCA and DCA drop. In the meantime, the total content of hydrophobic BAs decreases, and the TBA pool remains stable [67].

### Nervous system diseases

There are significant differences between intracerebral and extracerebral BAs compositions and concentrations. Mano et al. [91] conducted MPBA for the rat brain. CA, CDCA and DCA were distributed in the cytoplasm of the brain homogenate, of which CDCA accounted for 92.1%. In contrast, its content in blood was only 13.8%, which may be attributed to the synthesis pathway of BAs in the brain. The contents of BAs in blood gradually increase with the progression of cholestatic liver disease, triggering a series of detrimental events [92]. Taking obstructive cholestasis as the example, BAs in blood may enter the nervous system through the blood-brain barrier after reaching certain concentrations, further inducing neurotoxicity [93].

Hepatic encephalopathy (HE) is a brain dysfunction caused by metabolic disorders due to acute and chronic liver injuries [94]. In a rat model of cholestasis induced by biliary duct ligation complicated with HE, the LCA content in the brain significantly exceeds that of the sham operation group, so LCA may be a potential biomarker for diagnosing HE [95]. Cerebrotendinous xanthomatosis (CTX) occurs because cholesterol and cholestanol accumulate in the brain owing to abnormal lipid metabolism and decreased BAs synthesis induced by recessive inheritance of CYP27A1 gene [96]. CTX can be treated by BAs (including CDCA, UDCA, CA and TCA) replacement therapy that repairs BAs synthesis and reduces the level of cholestanol. Nevertheless, BAs concentrations in the brain should be moderate, so it is necessary to control BAs by monitoring their contents in blood [97].

## Conclusion

As signaling molecules that regulate lipid, glucose and energy metabolisms, BAs play important roles in many pathological and physiological activities. Accordingly, accurate monitoring of their contents is of great significance to both basic and clinical studies. Chromatographic technologies enable simultaneous qualitative and quantitative analyses, thus having become crucial strategies for BAs detection. With continuous development, the precision and sensitivity of chromatographic apparatuses have been gradually augmented, and a variety of BAs can now be separated and purified at ease. At present, both GC-MS and LC-MS can accurately separate and then quantify specific BAs. Regardless, since MS signals cannot distinguish BAs with conformational isomers, such as Tα-MCA, Tβ-MCA and Tω-MCA, these methods confront a bottleneck in complete separation. Furthermore, the process of sample treatment also markedly affects experimental results, such as attenuation of the matrix effect that is indispensable to the increase of precision and accuracy [98]. Hence, in the future, the degree of separation and sensitivity of chromatographic systems should be further improved, and more stable sample treatment methods are still in need.

The contents and compositions of different types of BAs vary largely during the progression of different diseases. Accordingly, researchers have endeavored to link MPBA with specific diseases, and to guide their diagnosis and treatment by monitoring the changes of BAs characteristics. Clinically, diseases are mainly diagnosed by detecting TBA in human serum, which is convenient, rapid and low-cost, with low specificity though [99]. In consideration of complicated procedures, high cost of apparatus and requirement of professional personnel, MPBA is now mainly applied in basic research but not clinical practice. This suggest that researchers should expedite the promotion of programmed automatic sample treatment methods in future studies, aiming to apply the monitoring of specific BAs in the diagnosis and treatment of clinical diseases. Meanwhile, unraveling the roles of MPBA in hepatobiliary, gastrointestinal, metabolic, neurological and other diseases will provide valuable guidance for the diagnosis and typing of related diseases such as ICP, or the diagnosis and prognosis of gastrointestinal cancer [100].

## Abbreviations

BAs: Bile acids
C4: 7α-hydroxy-4-cholesten-3-one
CA: Choli acid
CDCA: Chenodeoxycholic acid
CTX: Cerebrotendinous xanthomatosis
DCA: Deoxycholic acid
EI: Electron impact
ELISA: Enzyme-linked immunosorbent assay
ESI: Electrospray ionization
FGF15: Ibroblast growth factor 15
FXR: Farnesoid X receptor
GCA: Glycocholic acid
GCBAs: Glycine-conjugated BAs
GCDCA: Taurochenodeoxycholic acid
GC-MS: Gas chromatography
GDCA: Glycodeoxycholic acid
GDM: Gestational diabetes mellitus
GHDCA: Glycohyodeoxycholic acid
GLCA: Glycolithocholic acid
GUDCA: Glycoursodeoxycholic acid
HDCA: Hydeoxycholic acid
HE: Hepatic encephalopathy
HPLC: High performance liquid chromatography
ICP: Intrahepatic cholestasis of pregnancy
ILA: Isolithocholic acid
LCA: Lithocholic acid
LC-MS: Liquid chromatography-mass spectrometry
LLOQ: Lower limits of quantification
MDCA: Murodeoxycholic acid
MPBA: Metabolic profiling of BAs
MRM: Multiple reaction monitoring
MSTFA: N-methyl-N-(trimethylsilyl) trifluoroacetamide
NASH: Non-alcoholic steatohepatitis
NMR: Nuclear magnetic resonance
PXR: Pregnane X receptor
SCCO2: Supercritical carbon dioxide gas
SFC: Supercritical fluid chromatography
SIM: Single ion monitoring
SPE: Solid phase extraction
SPME: Solid phase microextraction
TBA: Total bile acid
TCA: Taurocholic acid
TCBAs: Taurine-conjugated BAs
TCDCA: Taurochenodeoxycholic acid
TDCA: Tauroursodeoxycholic acid
TFME: Choline-phospholipidsX
TFME: Thin-film microextraction
TGR5: G-protein-coupled BA receptor
THCA: Taurohyocholic acid
THDCA: Taurohyodeoxycholic acid
TLC: Layer chromatography
TUDCA: Tauroursodeoxycholic acid
Tβ-MCA: Tauro-β-muricholic acid
UDCA: Ursodeoxycholic acid
UFLC: Ultrafast LC
UPLC: Ultra-performance liquid chromatography
VDR: Vitamin D receptor
VLDL: Low density lipoprotein
α-MCA: α-muricholic acid
ω-MCA: ω-muricholic acid
